# Supplementary material for: Structural basis of metalloid transport by the arsenite efflux pump ArsB
Source: Nat Commun. 2026 May 18;17:6545. doi: 10.1038/s41467-026-73273-z (PMC13381914; doi:10.1038/s41467-026-73273-z)
Supplement: Supplementary file 1 — Supplementary Information [file 41467_2026_73273_MOESM1_ESM.pdf]

Supplementary information

Structural basis of metalloid transport by the arsenite efflux pump ArsB

Shivansh Mahajan[1,a], Kemal Demirer[2], William M. Clemons, Jr.[1,3,*] and Douglas C. Rees[1,*]

[1]Division of Chemistry and Chemical Engineering, [2]Division of Biology and Biological Engineering, California Institute of Technology, Pasadena, CA, USA; [3]Biohub, Redwood City, CA, USA.

[a]Present address: Department of Natural Product Biosynthesis, Max Planck Institute for Chemical Ecology, Jena, Germany 07745.

*Address correspondence to William M. Clemons, Jr. (clemons@caltech.edu) and Douglas C. Rees (dcrees@caltech.edu).

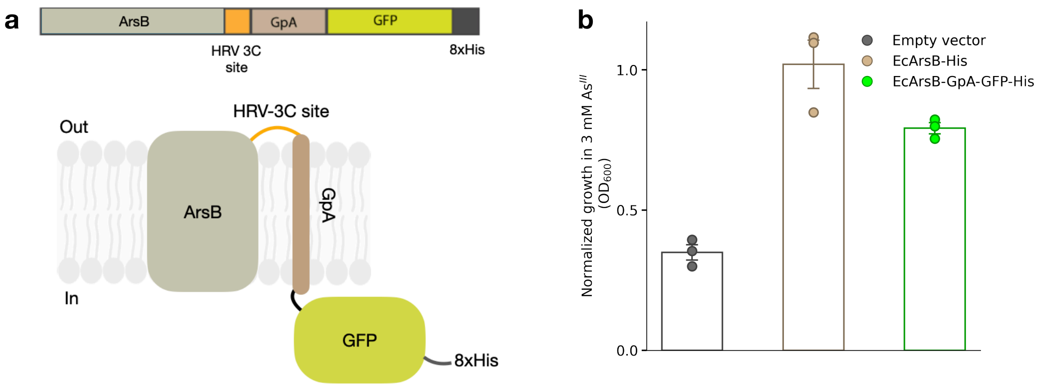

Supplementary Figure 1. *Lf*ArsB expression construct with GFP fusion. a Schematic representation of the *Lf*ArsB expression construct bearing a C-terminal fusion of HRV-3C protease cleavage site – glycophorin A (GpA) – GFP – 8xHis. **b** Normalized growths of *E. coli* AW3110 cells bearing *Ec*ArsB-His or EcArsB-GFP fusion construct (*Ec*ArsB-GpA-GFP-His) as described in **a**, in presence of 3 mM As^{III}. OD_{600} values in the presence of As^{III} are normalized by corresponding values in the absence of As^{III}. Biological triplicates (n = 3) are reported, and error bars represent standard error of mean. Source data for the assay are provided in the Source Data file.

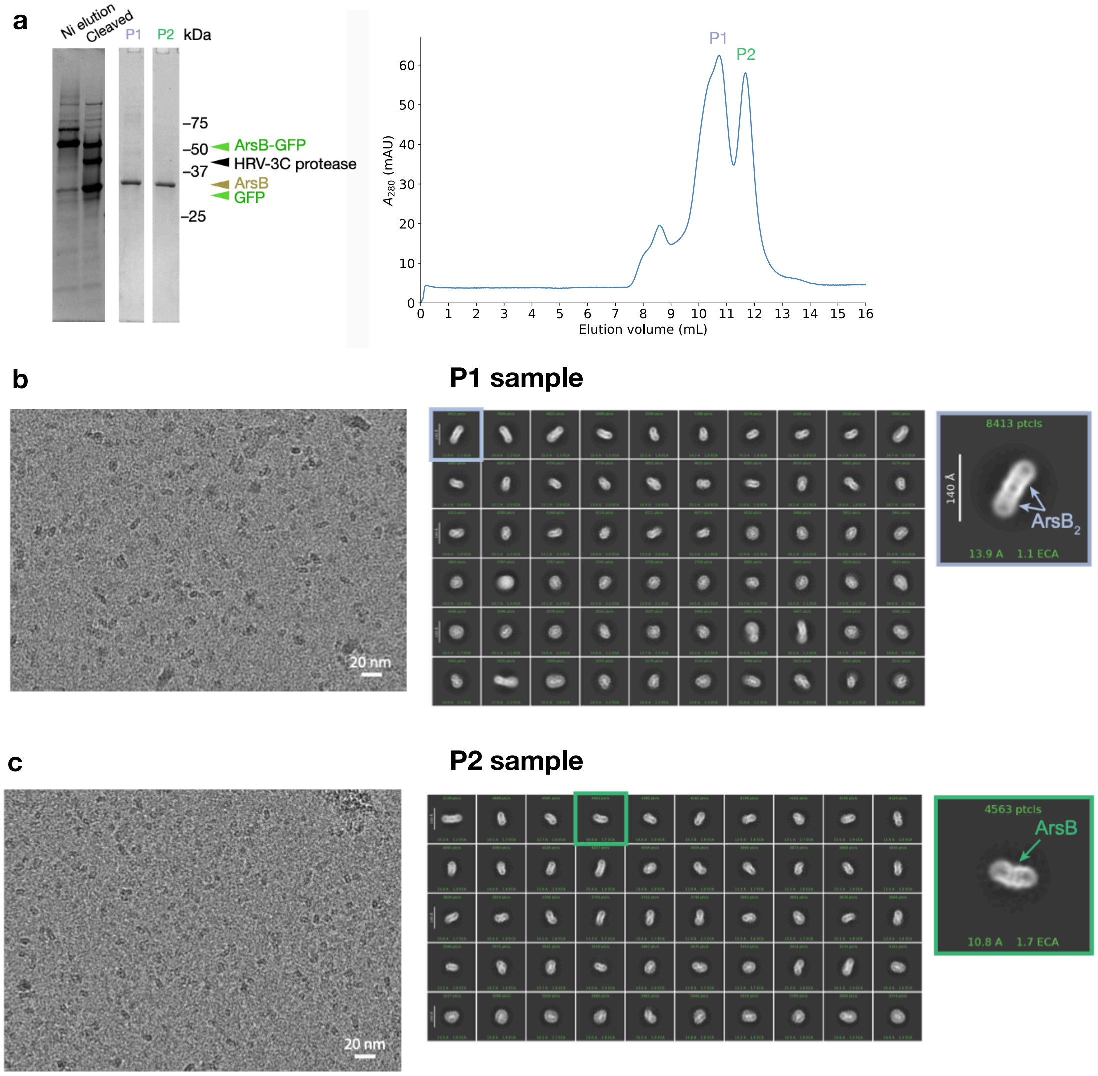

Supplementary Figure 2 . Purification and preliminary cryo-EM analysis of *Lf*ArsB solubilized in 0.03% (w/v) DDM. a SDS-PAGE analysis (left) and SEC profile (right). The P1 and P2 samples, which show a pure ArsB band on the gel, correspond to peaks P1 and P2 on the SEC trace. Representative micrograph at 130,000x magnification and 2D class averages corresponding to P1 and P2 samples are shown in panels **b** and **c**, respectively. P1 sample is composed of ArsB dimer ($ArsB_2$) in a micelle, whereas the P2 sample is composed of ArsB monomer in a micelle.

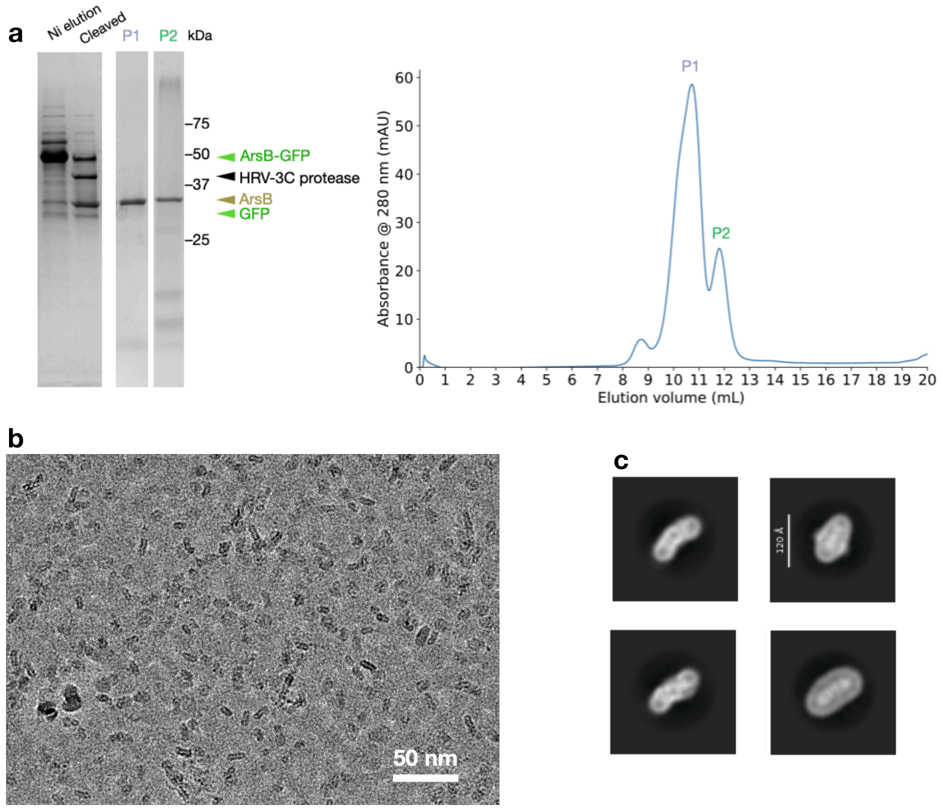

Supplementary Figure 3. Purification and preliminary cryo-EM analysis of *Lf*ArsB solubilized in 0.005%/0.0005% (w/v) LMNG/CHS. a SDS-PAGE analysis (left) and SEC profile (right). **b** Representative micrograph from P1 sample at 130,000x magnification. **c** Representative 2D class averages from P1 sample showing ArsB dimers in micelles.

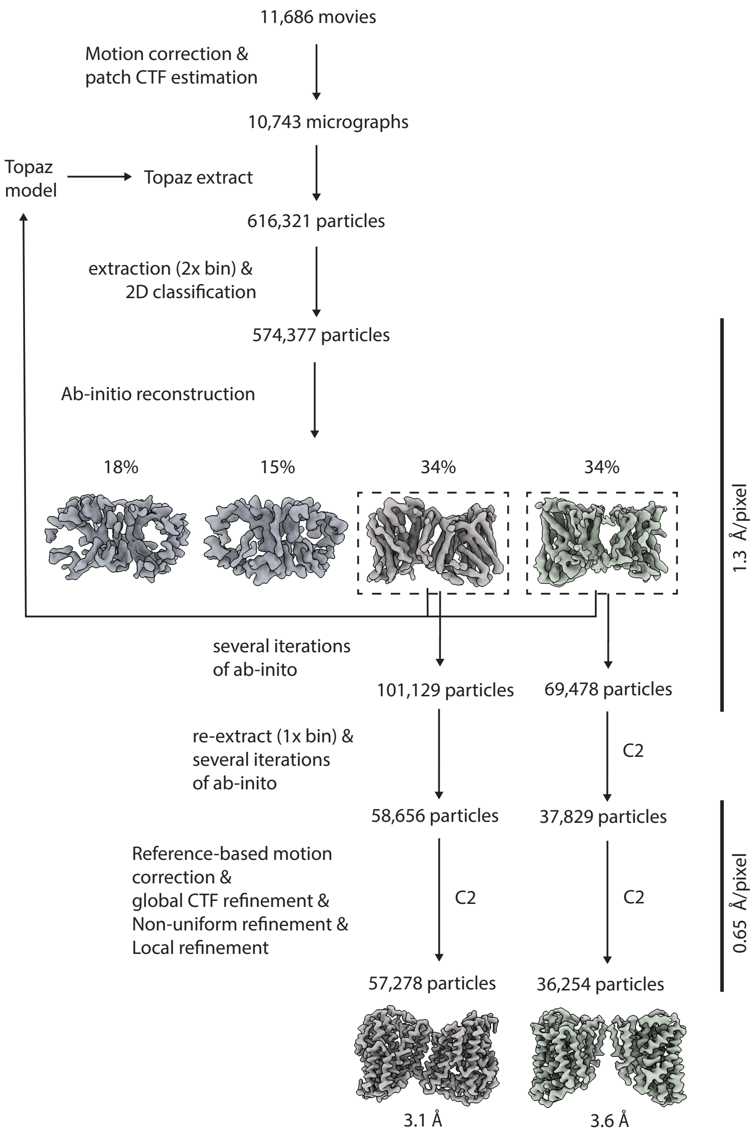

Supplementary Figure 4. Cryo-EM data processing workflow for apo *Lf*ArsB structures in cryoSPARC.

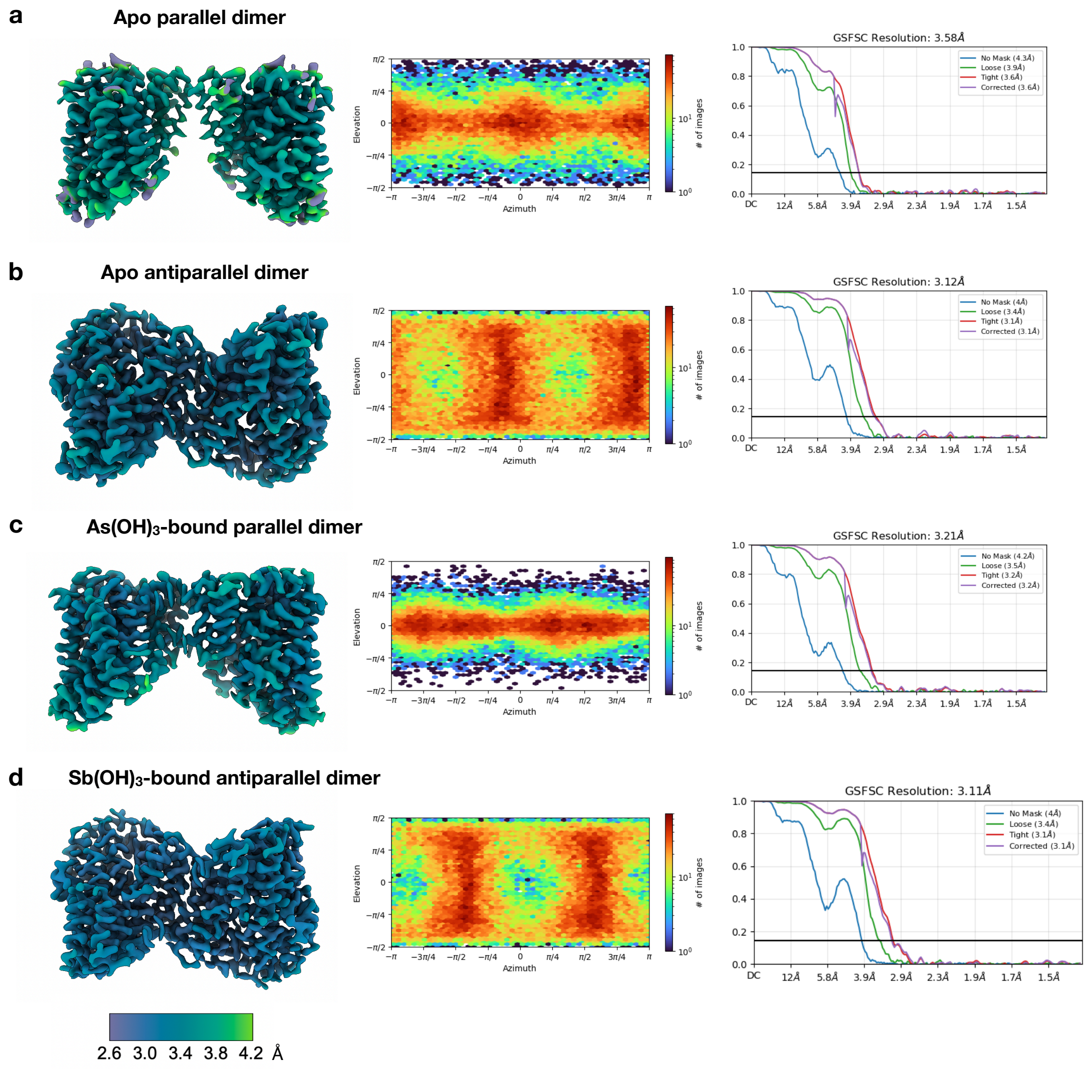

Supplementary Figure 5. Cryo-EM maps and validation for **a** apo *Lf*ArsB, **b** *Lf*ArsB + As(OH)$_3$, and **c** *Lf*ArsB + Sb(OH)$_3$. Left, B-factor sharpened map colored by local resolution; center, angular distribution heatmap plot; and right, gold-standard Fourier shell correlation (GSFSC) curve (cut-off: 0.143).

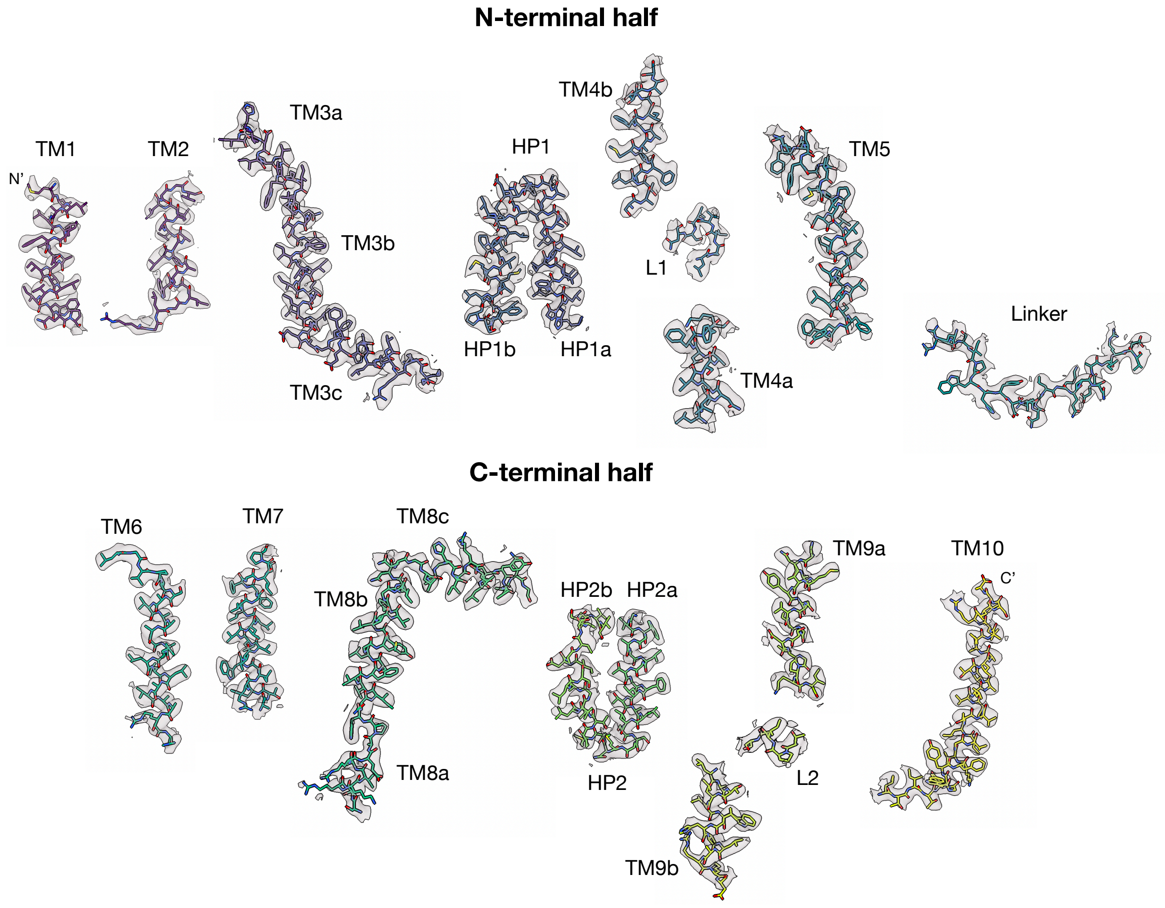

Supplementary Figure 6. Representative cryo-EM density maps for various segments of the apo antiparallel *Lf*ArsB structure. Model is shown as sticks and colored using the viridis palette.

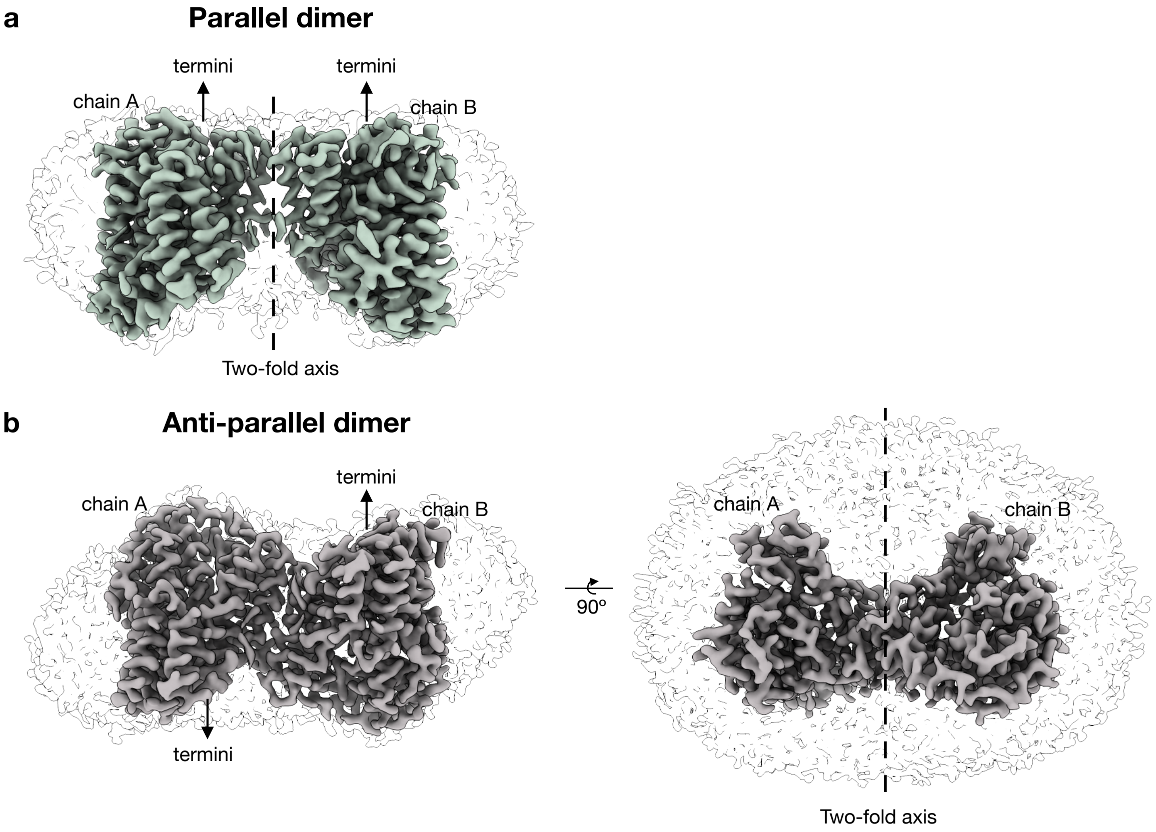

Supplementary Figure 7. Apo *Lf*ArsB dimer architectures in detergent micelles showing positions of the termini and the two-fold axis. a Parallel dimer. **b** Antiparallel dimer.

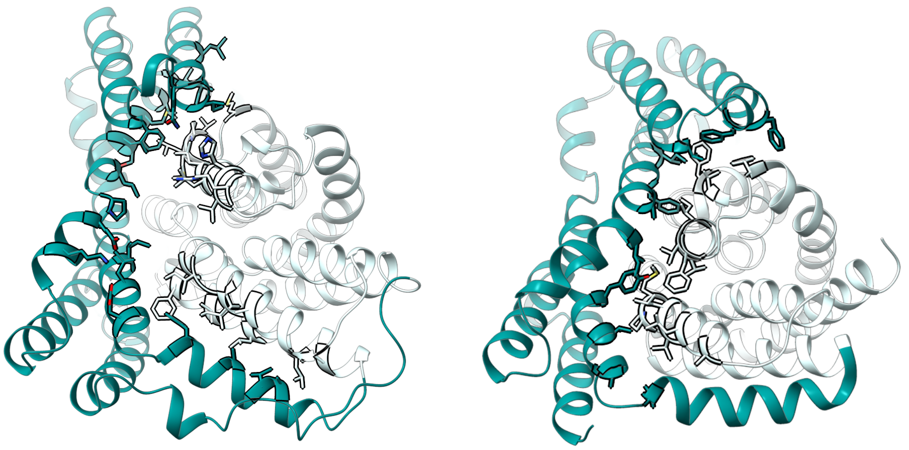

Supplementary Figure 8. Residues (shown as sticks) at the interface of transport domain (cyan) and scaffold domain (teal) of *Lf*ArsB in the cytoplasmic view (left) and periplasmic view (right).

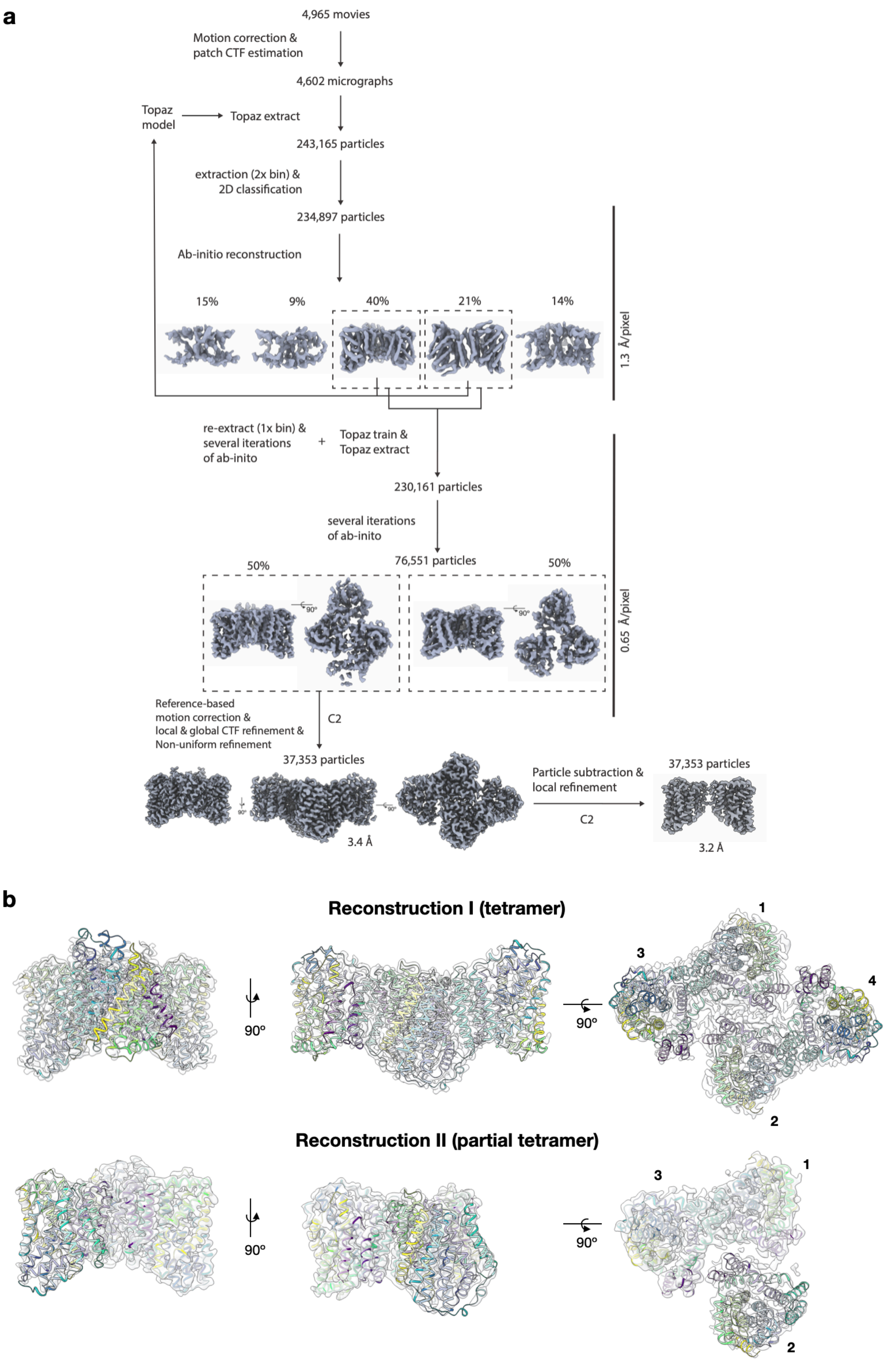

Supplementary Figure 9. Cryo-EM data processing for $As(OH)_3$-bound *Lf*ArsB structure. a Data processing workflow in cryoSPARC. **b** Two distinct reconstructions from this dataset with either three (reconstruction II) or four (reconstruction I) ArsB monomers. The final model of the $As(OH)_3$-bound structure was docked to show the composition of each reconstruction.

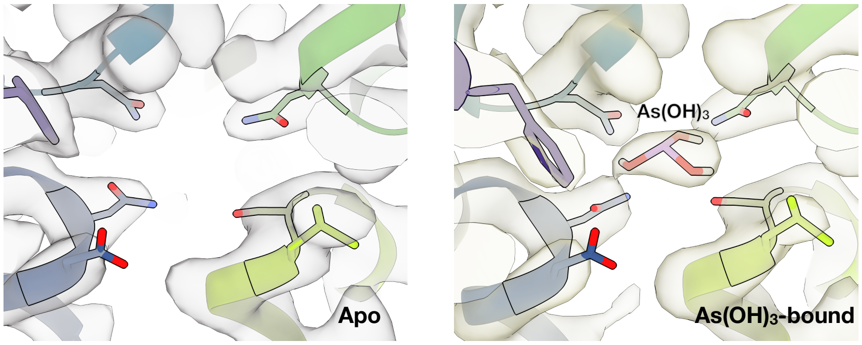

Supplementary Figure 10. Metalloid-binding site of *Lf*ArsB apo structure (left) and $As(OH)_3$-bound structure (right) and corresponding Coulomb potential maps normalized and contoured at a threshold level of 7.0.

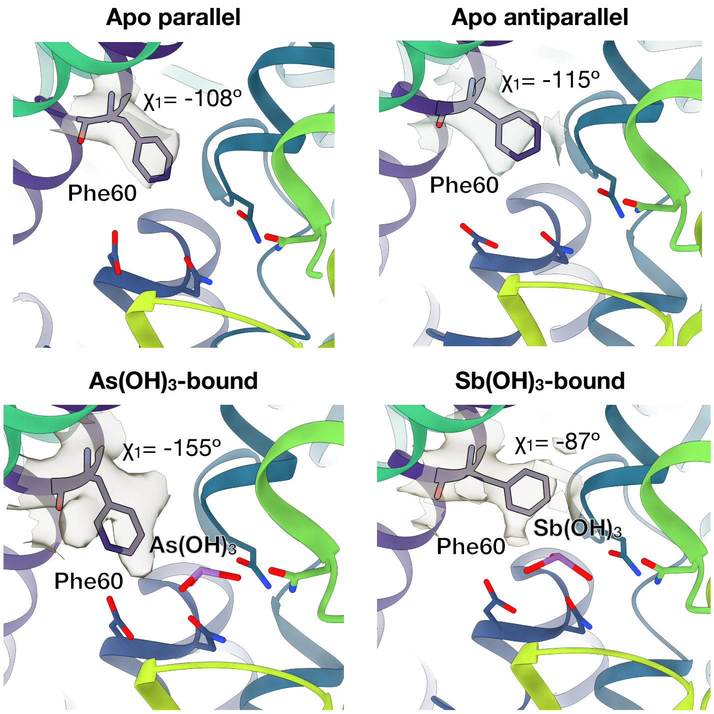

Supplementary Figure 11. Multiple conformations of Phe60 sidechain across apo and metalloid-bound *Lf*ArsB structures. Respective χ_1 torsion angles are labeled.

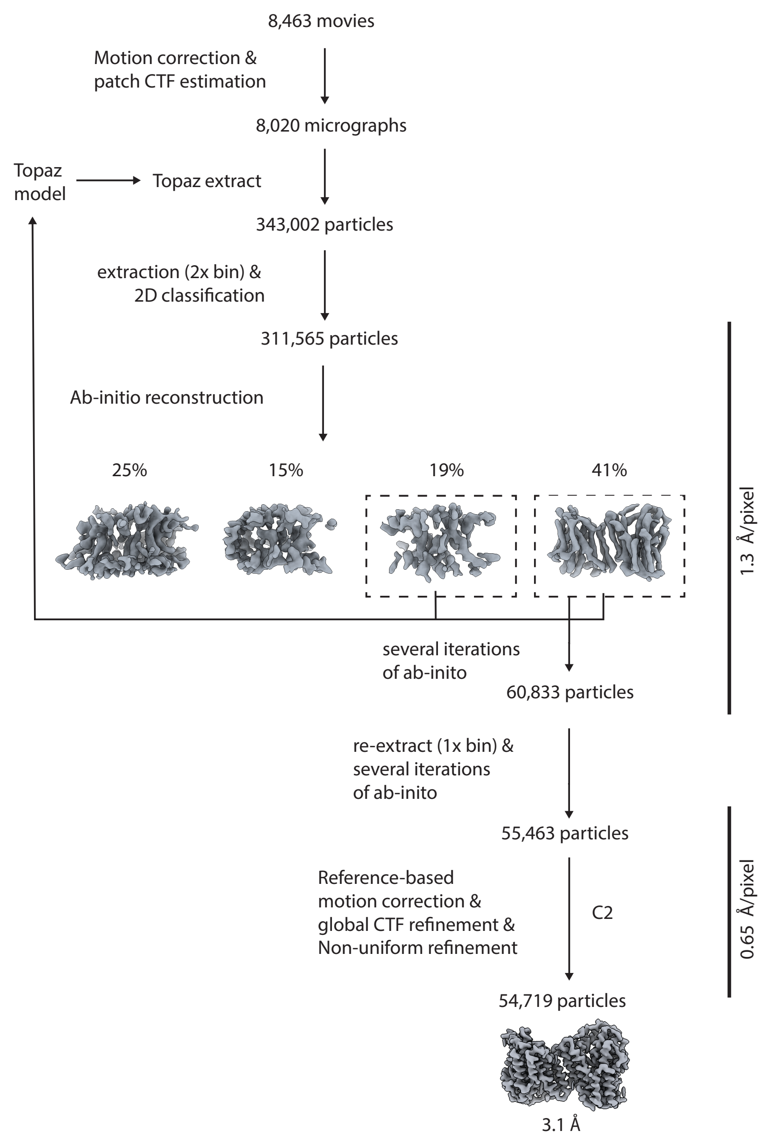

Supplementary Figure 12. Cryo-EM data processing workflow for $Sb(OH)_3$-bound *Lf*ArsB structure in cryoSPARC.

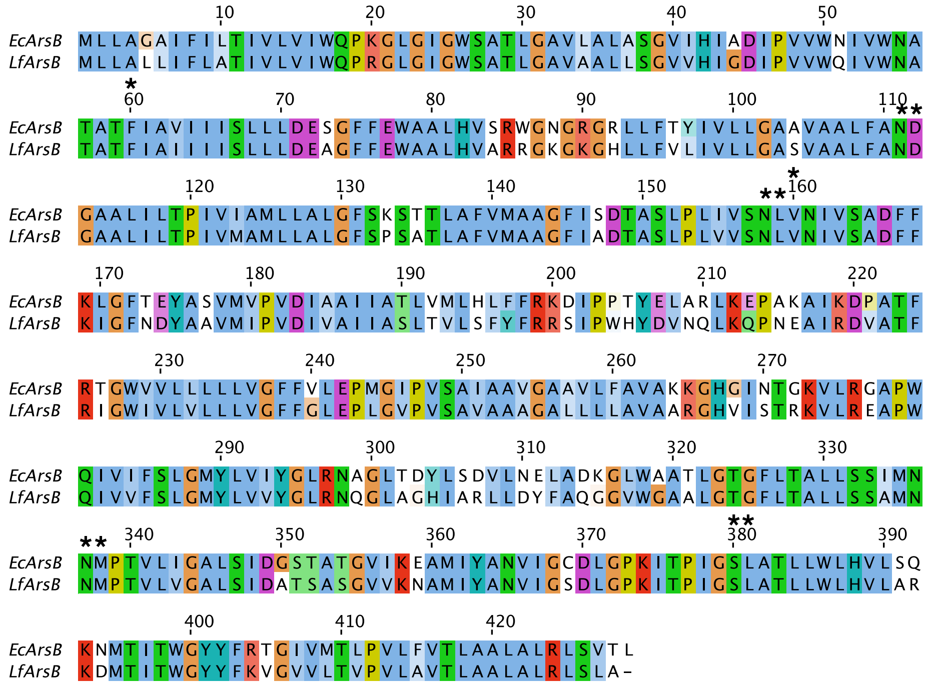

Supplementary Figure 13. Sequence alignment of *Ec*ArsB and *Lf*ArsB prepared and visualized using Jalview[1]. Conserved residues of the metalloid-binding pocket are indicated with an asterisk (*) above the residue.

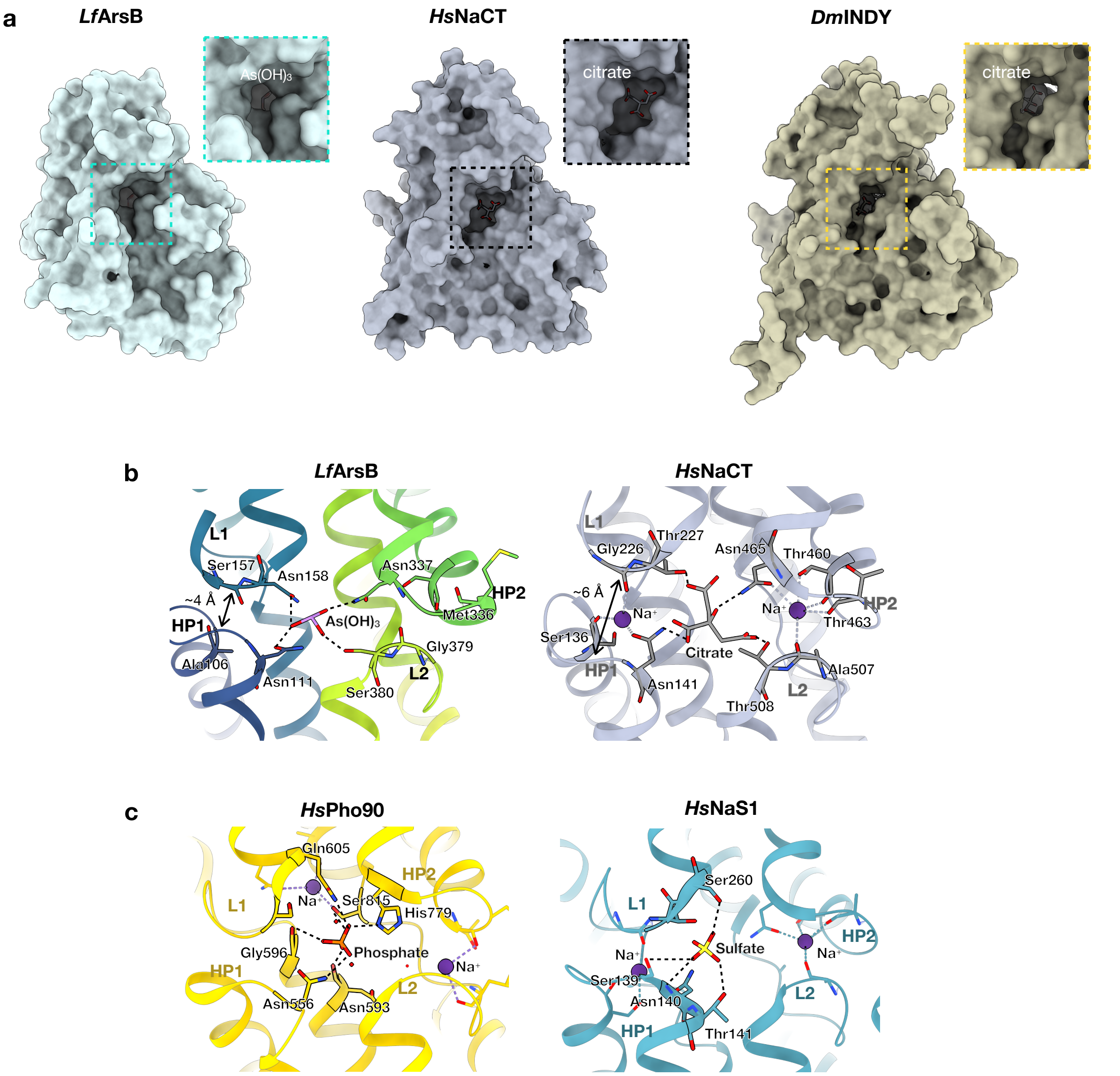

Supplementary Figure 14. Comparison of substrate-binding pockets of *Lf*ArsB (inward), *Hs*NaCT (inward-open), and *Dm*INDY (inward-occluded). a Surface representation of cytoplasmic view highlighting the size of the substrate-binding pocket in each structure. **b** Substrate interaction residues and helix-loop motif residues for Na^+ interaction in *Lf*ArsB and *Hs*NaCT. **c** Substrate recognition in a human low-affinity phosphate transporter, *Hs*Pho90 (PDB 8R34), and a human SLC13A1 sulfate transporter, *Hs*NaS1 (PDB 8Y5U).

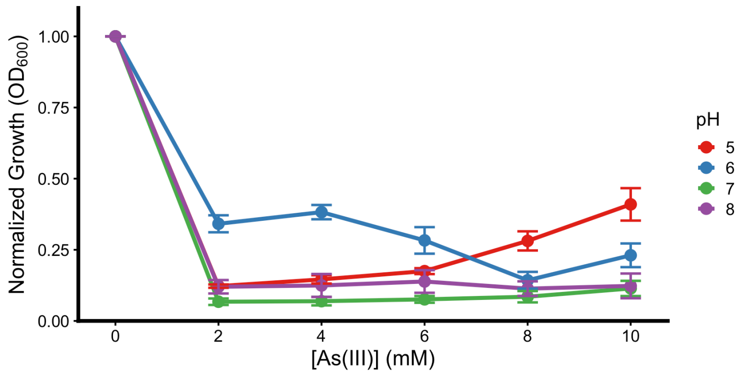

Supplementary Figure 15. External pH-dependence of As^{III} resistance conferred by empty pRSFdelT7 vector in AW3110 cells. Data points represent mean of biological quadruplicates (n = 4) and error bars represent standard error of mean. Source data for the assay are provided in the Source Data file.

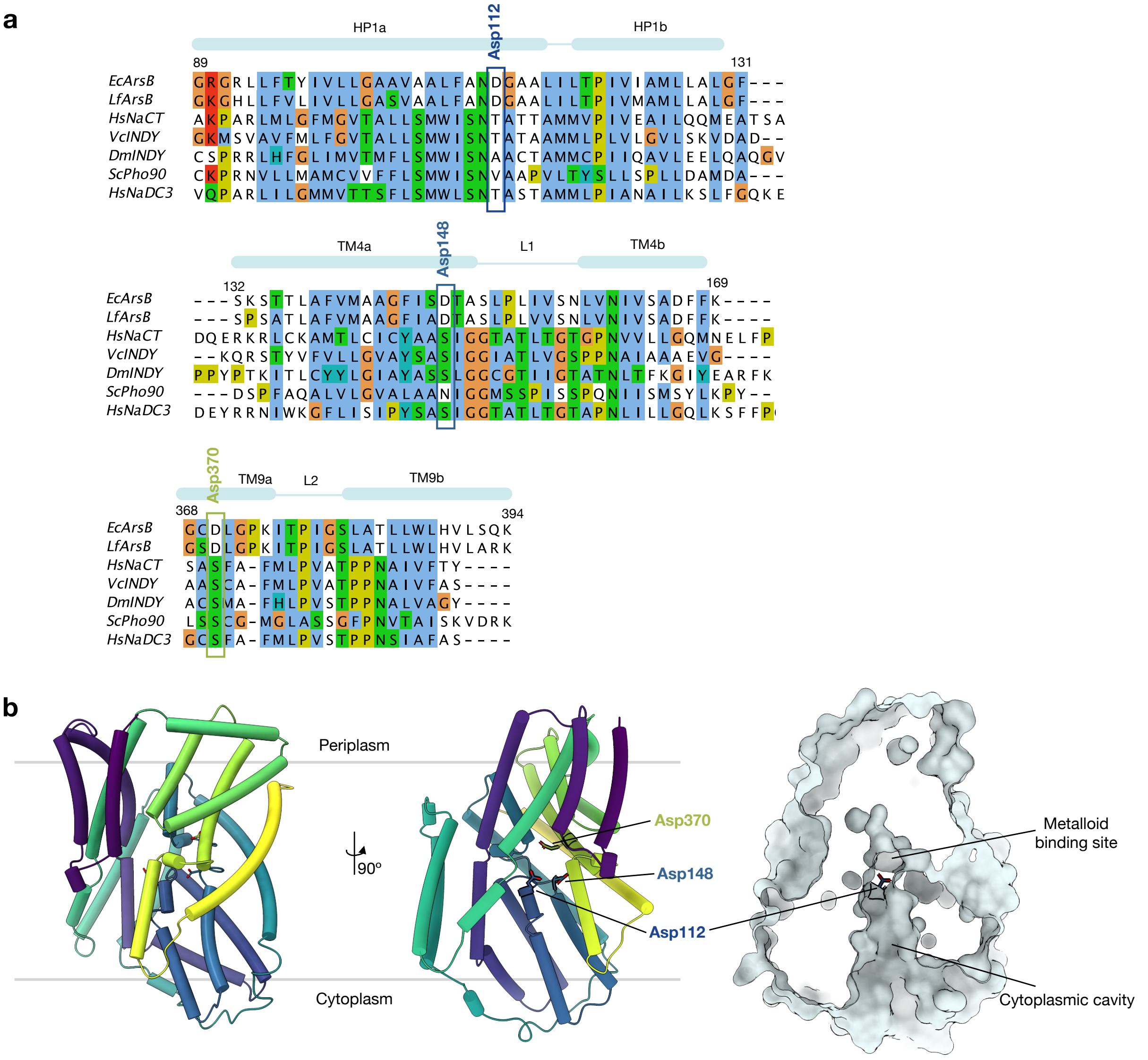

Supplementary Figure 16. H⁺-coupling mechanism of *Lf*ArsB. a Sequence alignment of ArsB and representative DASS transporters, highlighting that the putative H⁺-coupling Asp residues are not found beyond ArsB sequences. Alignment was prepared using structure-based sequence alignment (Promals3D[2]) and visualized in Jalview[1]. Representative sequence limits shown correspond to ArsB sequences. **b** Positions of H⁺-coupling Asp residues (Asp112, Asp148 and Asp370) in the *Lf*ArsB 'inward-facing' model.

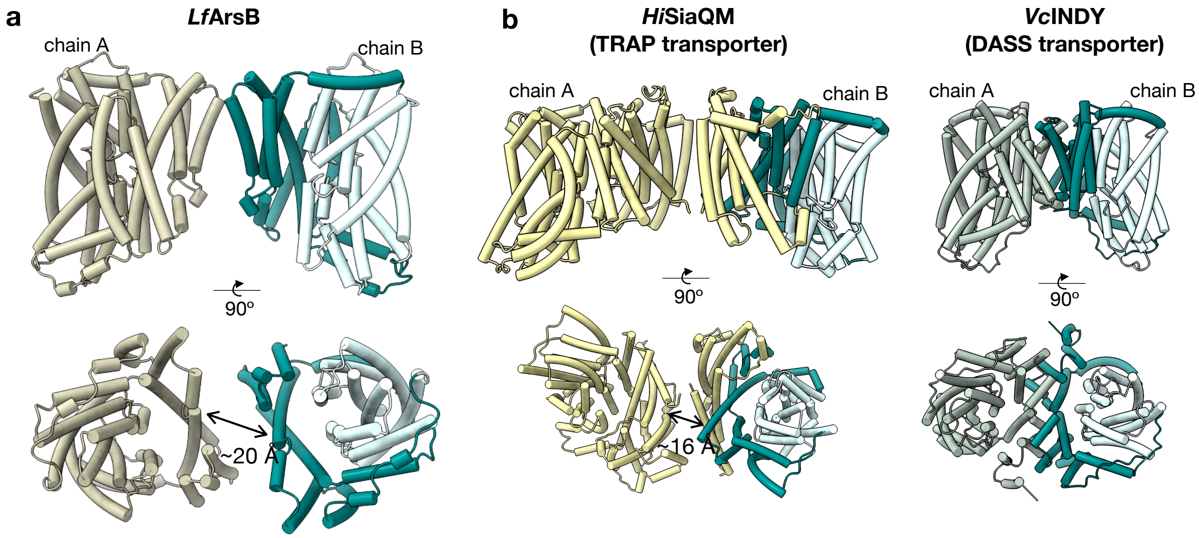

Supplementary Figure 17. Parallel dimers of **a** *Lf*ArsB, and **b** TRAP transporter, SiaQM, from *H. influenzae* (*Hi*SiaQM; PDB 8THI) and DASS transporter, *Vc*INDY (PDB 7T9F), in two orientations.

Supplementary Table 1. Cryo-EM data collection, refinement and validation statistics.

	*Lf*ArsB apo parallel dimer (PDB ID 10TP)	*Lf*ArsB apo antiparallel dimer (PDB ID 10TQ)	*Lf*ArsB + As^{III} parallel dimer (PDB ID 10TU)	*Lf*ArsB + Sb^{III} antiparallel dimer (PDB ID 10UA)
**Data collection and processing**				
Magnification	130,000	130,000	130,000	130,000
Voltage (kV)	300	300	300	300
Electron exposure ($e^{–}/Å^2$)	70	70	70	70
Defocus range (μm)	-0.8 to -2.8	-0.8 to -2.8	-0.8 to -2.8	-0.8 to -2.8
Pixel size (Å) (Super-resolution mode)	0.325	0.325	0.325	0.325
Movies	11,686	11,686	4,965	8,463
Total extracted particles	616,321	616,321	316,729	343,002
Final particles	57,278	36,254	37,353	54,719
Symmetry imposed	C2	C2	C2	C2
Map resolution (Å) (FSC 0.143 cut-off)	3.6	3.1	3.2	3.1
**Refinement**				
Initial model used	*Lf*ArsB AlphaFold model	*Lf*ArsB AlphaFold model	*Lf*ArsB AlphaFold model	*Lf*ArsB AlphaFold model
Model resolution (Å)	3.9	3.4	3.5	3.4
Map sharpening *B* factor ($Å^2$)	-117	-112	-92	-119
Model composition:				
Protein residues	856	856	856	856
Ligands			$As(OH)_3$	$Sb(OH)_3$
Model B factors ($Å^2$):				
Protein	61	67	70	81
Ligand			101	123
R.m.s. deviations:				
Bond lengths (Å)	0.007	0.008	0.008	0.007
Bond angles (°)	0.687	0.703	0.603	0.587
**Validation**				
MolProbity score	1.30	1.30	1.46	1.54
Clashscore	5.61	5.53	5.68	5.68
Poor rotamers (%)	0	0	0	0
Ramachandran plot:				
Favored (%)	98.4	98.4	97.2	96.5
Allowed (%)	1.6	1.6	2.8	3.5
Outliers (%)	0	0	0	0

References

1. Waterhouse, A. M., Procter, J. B., Martin, D. M. A., Clamp, M. & Barton, G. J. Jalview Version 2—a multiple sequence alignment editor and analysis workbench. *Bioinformatics* **25**, 1189–1191 (2009).
2. Pei, J., Kim, B.-H. & Grishin, N. V. PROMALS3D: a tool for multiple protein sequence and structure alignments. *Nucleic Acids Research* **36**, 2295–2300 (2008).
