# Supplementary material for: Structural basis of metalloid transport by the arsenite efflux pump ArsB
Source: Nat Commun. 2026 May 18;17:6545. doi: 10.1038/s41467-026-73273-z (PMC13381914; doi:10.1038/s41467-026-73273-z)
Supplement: Supplementary file 2 — Reporting Summary [file 41467_2026_73273_MOESM2_ESM.pdf]

nature portfolio

Corresponding author(s): William M. Clemons Jr. & Douglas C. Rees

Last updated by author(s): April 11, 2026

Reporting Summary

Nature Portfolio wishes to improve the reproducibility of the work that we publish. This form provides structure for consistency and transparency in reporting. For further information on Nature Portfolio policies, see our Editorial Policies and the Editorial Policy Checklist.

Statistics

For all statistical analyses, confirm that the following items are present in the figure legend, table legend, main text, or Methods section.

n/a	Confirmed	
☐	☒	The exact sample size (*n*) for each experimental group/condition, given as a discrete number and unit of measurement
☐	☒	A statement on whether measurements were taken from distinct samples or whether the same sample was measured repeatedly
☒	☐	The statistical test(s) used AND whether they are one- or two-sided<br>*Only common tests should be described solely by name; describe more complex techniques in the Methods section.*
☒	☐	A description of all covariates tested
☒	☐	A description of any assumptions or corrections, such as tests of normality and adjustment for multiple comparisons
☐	☒	A full description of the statistical parameters including central tendency (e.g. means) or other basic estimates (e.g. regression coefficient) AND variation (e.g. standard deviation) or associated estimates of uncertainty (e.g. confidence intervals)
☒	☐	For null hypothesis testing, the test statistic (e.g. *F*, *t*, *r*) with confidence intervals, effect sizes, degrees of freedom and *P* value noted<br>*Give P values as exact values whenever suitable.*
☒	☐	For Bayesian analysis, information on the choice of priors and Markov chain Monte Carlo settings
☒	☐	For hierarchical and complex designs, identification of the appropriate level for tests and full reporting of outcomes
☒	☐	Estimates of effect sizes (e.g. Cohen's *d*, Pearson's *r*), indicating how they were calculated

Our web collection on statistics for biologists contains articles on many of the points above.

Software and code

Policy information about availability of computer code

Data collection	EM data collection: SerialEM 4.1.1<br>arsenite resistance growth assays: BioTek Gen5 v3.16
Data analysis	EM data processing: cryoSPARC v4.7.1, Topaz 0.2.5a<br>Model building and refinement: ChimeraX v1.10, Phenix v1.21.2, Coot v0.9.8.7<br>Growth data analysis: RStudio

For manuscripts utilizing custom algorithms or software that are central to the research but not yet described in published literature, software must be made available to editors and reviewers. We strongly encourage code deposition in a community repository (e.g. GitHub). See the Nature Portfolio guidelines for submitting code & software for further information.

Data

Policy information about availability of data

All manuscripts must include a data availability statement. This statement should provide the following information, where applicable:

- Accession codes, unique identifiers, or web links for publicly available datasets
- A description of any restrictions on data availability
- For clinical datasets or third party data, please ensure that the statement adheres to our policy

Atomic coordinates for all the structures reported in the paper are accessible on the Protein Data Bank (PDB) using the accession codes: 10TP (apo parallel dimer),

10TQ (apo antiparallel dimer), 10TU (As(OH)3-bound parallel dimer), and 10UA (Sb(OH)3-bound antiparallel dimer). Corresponding EM density maps can be accessed on the Electron Microscopy Data Bank (EMDB) using accession codes: EMD-75462 (apo parallel dimer), EMD-75463 (apo antiparallel dimer), EMD-75467 (As(OH)3-bound parallel dimer) and EMD-75468 (Sb(OH)3-bound antiparallel dimer). Additionally, EM density maps for tetramer and partial tetramer obtained from the As(OH)3 dataset have been deposited on the EMDB and can be accessed using codes EMD-76265 and EMD-76271, respectively. Source data for the AsIII resistance growth assay plots is provided as well.

Research involving human participants, their data, or biological material

Policy information about studies with human participants or human data. See also policy information about sex, gender (identity/presentation), and sexual orientation and race, ethnicity and racism.

Reporting on sex and gender	NA
Reporting on race, ethnicity, or other socially relevant groupings	NA
Population characteristics	NA
Recruitment	NA
Ethics oversight	NA

Note that full information on the approval of the study protocol must also be provided in the manuscript.

Field-specific reporting

Please select the one below that is the best fit for your research. If you are not sure, read the appropriate sections before making your selection.

[x] Life sciences   [ ] Behavioural & social sciences   [ ] Ecological, evolutionary & environmental sciences

For a reference copy of the document with all sections, see nature.com/documents/nr-reporting-summary-flat.pdf

Life sciences study design

All studies must disclose on these points even when the disclosure is negative.

Sample size	No sample-size calculations were performed. Cryo-EM data collection was constrained by microscope access and collection time. For growth data, four biological replicates were collected.
Data exclusions	As a standard practice in single particle cryo-EM data processing, poor quality micrographs and particles were excluded during data processing pipeline to achieve high-resolution reconstruction.
Replication	Arsenite resistance growth experiments were conducted in quadruplicates (n=4) of independent biological samples. All attempts at replication were successful.
Randomization	Randomization was not performed as randomization is not relevant to the structural and biochemical data reported in this study. Sample allocation does not affect the experimental data in this study.
Blinding	Blinding was not performed as the data reported in this study does not require subject assessment that may influence the validity of the results.

Reporting for specific materials, systems and methods

We require information from authors about some types of materials, experimental systems and methods used in many studies. Here, indicate whether each material, system or method listed is relevant to your study. If you are not sure if a list item applies to your research, read the appropriate section before selecting a response.

Materials & experimental systems

n/a	Involved in the study	
x		Antibodies
x		Eukaryotic cell lines
x		Palaeontology and archaeology
x		Animals and other organisms
x		Clinical data
x		Dual use research of concern
x		Plants

Methods

n/a	Involved in the study	
x		ChIP-seq
x		Flow cytometry
x		MRI-based neuroimaging

Plants

Seed stocks	NA
Novel plant genotypes	NA
Authentication	NA
