# Supplementary material for: Structural basis of metalloid transport by the arsenite efflux pump ArsB
Source: Nat Commun. 2026 May 18;17:6545. doi: 10.1038/s41467-026-73273-z (PMC13381914; doi:10.1038/s41467-026-73273-z)
Supplement: Supplementary file 3 — Transparent Peer Review file [file 41467_2026_73273_MOESM3_ESM.pdf]

nature portfolio

Peer Review File

Structural basis of metalloid transport by the arsenite efflux pump ArsB

Corresponding Author: Professor William Clemons

This file contains all reviewer reports in order by version, followed by all author rebuttals in order by version.

Version 0:

Reviewer comments:

Reviewer #1

(Remarks to the Author)
The study by Mahajan et al. reports the first structures of the hydroxylated metalloid exporter ArsB. The authors use CryoEM in combination with cellular uptake assays of ArsB mutants to interrogate the protein's substrate-proton coupling.

The study appears well-conducted, the analysis is appropriate, and the results will be exciting to the transporter community. In particular, I expect significant interest in this structure in deciphering DASS transport mechanism(s) and substrate selectivity.

There are a few modest bits of data and points of comparison that the authors should include for completeness in this study. Nevertheless, I am confident these are easily achievable, and the manuscript will ultimately be made acceptable for publication in Nature Communications.

- The nomenclature of the substrates is a bit confusing. Noting the oxidation state of the metal can give the impression that LfArsB is a metal transporter. Furthermore, using the name (arsenite) appears incorrect, as the author notes that only the neutral trihydroxylated form is present at physiological pH. Rather, I believe the correct name for this substrate is "arsenous acid" and suggest using this or the formula throughout for clarity.
- The authors should compare their substrate-binding mode and selectivity mechanism with those of related IT transporters with inorganic substrates: SLC13A1 and Pho90.
- Growth assays are an indirect measurement of proton coupling and may be confounded by other physiological processes. The authors should verify their expected proton-coupling mechanism (and ideally probe the residues involved) using in vitro measurements on purified protein.
- In a few places, the authors state that DASS transporters "typically" use a sodium-substrate co-transport mechanism. However, this is an over-generalization, perhaps based on the characterization of human proteins or homologs of these genes. The sodium-independent exchange of carboxylate substrates is well-known for the DASS-Exchanger clade of genes in plants and bacteria, including biochemical characterization and structures of these proteins.
- The authors speculate in the discussion that H+ release and metalloid binding may be coupled, but it is unclear whether this is a general statement or whether there are specific structural insights to support this hypothesis. This is a key question in the IT superfamily and should be discussed with clarity and detail (if possible).
- Strong suggestion: Deposit the tetramer map and model. This will prove a valuable reference point for the field when studying detergent effects on the oligomeric states of membrane proteins.
- Relatedly, the supplementary figure 9 (data processing of $As(OH)_3$-bound LfArsB) appears to show a trimeric species that was reasonably well resolved. Is this modelable? And if so, I suggest this be deposited as well.

Reviewer #2

(Remarks to the Author)
The manuscript by Mahajan and colleagues reports structural and mechanistic characterization of the arsenite efflux pump ArsB from the arsenite-resistant bacterium L. ferriphilium. Being highly toxic, arsenite is found throughout the environment. Understanding the resistance mechanism in bacteria may lead future applications in bioengineering and environmental science. This work is novel in several aspects. By determining the cryo-EM structures of ArsB in apo and substrate-bound

states, the authors elucidate the characteristics of the substrate binding site for the neutral substrates. In combination with mutagenesis and transport assays in the cell, they further studied the coupling mechanism of the antiport proton motive force and identified two aspartate residues involved in protonation. All these, along with the oligomeric state of transporter, make ArsB distinct from the previously-characterized DASS family of proteins. The experiments described in the manuscript were carefully carried out, the text was clearly written and the figures well prepared. The work should certainly be seen by the broad readership of Nature Communications.

Here are some minor comments:

1. The authors model that ArsA transfers a substrate to ArsB and helps to increase the transport efficiency is intriguing. Perhaps, the authors would like to include a schematic drawing to illustrate the mechanism?
2. The style of the references cited is inconsistent; for some of them the information is incomplete.
3. In Table S1, the model resolution and the B-factor for the model should be included.

Open Access This Peer Review File is licensed under a Creative Commons Attribution 4.0 International License, which permits use, sharing, adaptation, distribution and reproduction in any medium or format, as long as you give appropriate credit to the original author(s) and the source, provide a link to the Creative Commons license, and indicate if changes were made.
In cases where reviewers are anonymous, credit should be given to 'Anonymous Referee' and the source.
The images or other third party material in this Peer Review File are included in the article’s Creative Commons license, unless indicated otherwise in a credit line to the material. If material is not included in the article’s Creative Commons license and your intended use is not permitted by statutory regulation or exceeds the permitted use, you will need to obtain permission directly from the copyright holder.
To view a copy of this license, visit https://creativecommons.org/licenses/by/4.0/

We thank the reviewers for their supportive and constructive feedback on our manuscript. We provide detailed responses to the comments and have revised the corresponding sections in the manuscript. Changes to the main text and supplementary information are marked with track changes. To address points #2, 6, and 7 from Reviewer #1, we have added two additional figure panels to the supplementary information – SI Fig. 9b and Fig. 14c. Included the source data for the growth assays in an Excel spreadsheet and added a note in the 'Data availability' section. Our responses follow, highlighted in blue.

Reviewer #1 Comments:

The study by Mahajan et al. reports the first structures of the hydroxylated metalloid exporter ArsB. The authors use CryoEM in combination with cellular uptake assays of ArsB mutants to interrogate the protein's substrate-proton coupling.

The study appears well-conducted, the analysis is appropriate, and the results will be exciting to the transporter community. In particular, I expect significant interest in this structure in deciphering DASS transport mechanism(s) and substrate selectivity.

There are a few modest bits of data and points of comparison that the authors should include for completeness in this study. Nevertheless, I am confident these are easily achievable, and the manuscript will ultimately be made acceptable for publication in Nature Communications.

1. The nomenclature of the substrates is a bit confusing. Noting the oxidation state of the metal can give the impression that LfArsB is a metal transporter. Furthermore, using the name (arsenite) appears incorrect, as the author notes that only the neutral trihydroxylated form is present at physiological pH. Rather, I believe the correct name for this substrate is "arsenous acid" and suggest using this or the formula throughout for clarity.

   Arsenite [As(III) or As^{III}] has been widely used in the arsenic detoxification literature, mostly to denote the trivalent state of arsenic without specifying its speciation in solution. Although the aqueous species of arsenite has been experimentally shown to be $As(OH)_3$, also called arsenous acid, the latter can undergo rapid ligand substitution in other cysteine-rich arsenic-binding proteins such as ArsA and ArsD to form tri-coordinate As^{III}-S bonds, suggesting a dynamic nature of arsenite speciation. This property highlights the metallic and non-metallic characteristics of arsenic, which may lead to confusion with its nomenclature. Indeed, the corresponding anhydrous salt of arsenite – sodium arsenite ($NaAsO_2$) – undergoes hydration upon dissolution in water to form $As(OH)_3$.

   While ArsB is not a metal transporter in the strict sense, the oxidation state of the metalloid substrate is an important feature of the detoxification process. However, we agree with the reviewer's concern about using arsenite or As^{III} to refer to the species interacting with ArsB. Therefore, when referring to the ArsB-bound species,

we have used $As(OH)_3$ to clearly define the transporter-bound state of the metalloid, which is supported by the interactions observed between the metalloid and the binding site residues. Nevertheless, to keep the nomenclature consistent with other studies on arsenic detoxification, we have used the term As^{III} to refer to arsenite, as in the growth assays. We believe the suggested usage of "arsenous acid", though chemically correct, may potentially lead to confusion and inconsistency with the literature.

2. The authors should compare their substrate-binding mode and selectivity mechanism with those of related IT transporters with inorganic substrates: SLC13A1 and Pho90.

   To incorporate this comparison, we have added a panel to SI Figure 14 (Supplementary Fig. 14c), showing the substrate-binding sites of SLC13A1 (sulfate transporter) and Pho90 (low-affinity phosphate transporter). Comparison of the substrate binding mode has been made in '*Structural comparison of ArsB with DASS family transporters'* sub-section of the '*Results'* (lines 232-240). Correspondingly, substrate binding across IT proteins is further discussed in *'Discussion'* (lines 314-321).

3. Growth assays are an indirect measurement of proton coupling and may be confounded by other physiological processes. The authors should verify their expected proton-coupling mechanism (and ideally probe the residues involved) using in vitro measurements on purified protein.

   We agree that conducting *in vitro* transport measurements using purified ArsB is an important next step for further validating the model. These experiments are complex and beyond the scope of what we can undertake as part of revising this manuscript. Based on our experience with ABC transporters, *in vitro* reconstitution of transport activity is a substantial research project in itself, involving challenges related to reconstitution protocols, transporter orientation in the bilayer, and potential lipid-specific effects. Additionally, a minor complication is that the first author has moved on to a postdoctoral position abroad and is unable to perform this work.

   To address this, we have added a note in the Discussion section (lines 339-341) about in vitro transport assays. "A next step to validate this model would involve in vitro metalloid transport assays using liposomes reconstituted with ArsB to enable direct measurement of ΔpH dependence and the H+-coupling mechanism of ArsB."

4. In a few places, the authors state that DASS transporters "typically" use a sodium-substrate co-transport mechanism. However, this is an over-generalization, perhaps based on the characterization of human proteins or homologs of these genes. The sodium-independent exchange of carboxylate substrates is well-known for the DASS-Exchanger clade of genes in plants and bacteria, including biochemical characterization and structures of these proteins.

We appreciate the reviewer for pointing this out regarding DASS co-transporter mechanism. We have clarified the corresponding statement in the *'Introduction'* (lines 58-59) to reference the DASS-exchanger proteins. Also, in the '*Structural comparison of ArsB with DASS family transporters'* sub-section of the '*Results'* (line 244), we have replaced "typically" with "symporters" to refer to only the DASS symporters that use Na^+-ion coupling.

5. The authors speculate in the discussion that H+ release and metalloid binding may be coupled, but it is unclear whether this is a general statement or whether there are specific structural insights to support this hypothesis. This is a key question in the IT superfamily and should be discussed with clarity and detail (if possible).

   While our inward-facing structure is consistent with H^+ release and metalloid binding from the cytoplasm, it does not provide any insight into the coupling of the two events. We used this statement in the '*Discussion'* (lines 343-345) to speculate about the mechanistic model of transport. For clarity, we have modified the corresponding sentence and noted that the coupling of the two events remains unclear.

6. Strong suggestion: Deposit the tetramer map and model. This will prove a valuable reference point for the field when studying detergent effects on the oligomeric states of membrane proteins.

   We agree with the value of providing this data, and the tetramer cryo-EM map has been deposited in the EMDB under the ID, EMD-76265. While the tetramer density is clear, the additional subunits are poorly resolved compared to the subunits in the parallel dimer (used for focused refinement) and are likely to cause confusion if we provide a structural model. We believe that the cryo-EM map of the tetrameric complex will be sufficient to demonstrate detergent effects on membrane protein oligomerization.

7. Relatedly, the supplementary figure 9 (data processing of As(OH)3-bound LfArsB) appears to show a trimeric species that was reasonably well resolved. Is this modelable? And if so, I suggest this be deposited as well.

   The trimeric species observed in the 3D classification of the $As(OH)_3$-bound dataset is likely a sub-population of the tetramer reconstruction. In this case, the well-resolved components are the subunits making up the antiparallel dimer, whereas the third potential subunit is not. The fourth subunit is likely an average of two alternate-facing orientations, which are not visible. As in the response to comment #6, we decided not to model into the 'trimer' map. We have deposited the corresponding map to the EMDB under the ID, EMD-76271. To prevent confusion with biological assemblies in the membrane protein field, we avoided using the terms 'tetramer' and 'trimer' for these oligomers in the deposited maps, instead referring to them as 'reconstruction I' and 'reconstruction II', respectively.

We have added a supplementary figure (Supplementary Fig. 9b) showing the composition of the two reconstructions before focused refinement.

Reviewer #2 Comments:

The manuscript by Mahajan and colleagues reports structural and mechanistic characterization of the arsenite efflux pump ArsB from the arsenite-resistant bacterium L. ferriphilium. Being highly toxic, arsenite is found throughout the environment. Understanding the resistance mechanism in bacteria may lead future applications in bioengineering and environmental science. This work is novel in several aspects. By determining the cryo-EM structures of ArsB in apo and substrate-bound states, the authors elucidate the characteristics of the substrate binding site for the neutral substrates. In combination with mutagenesis and transport assays in the cell, they further studied the coupling mechanism of the antiport proton motive force and identified two aspartate residues involved in protonation. All these, along with the oligomeric state of transporter, make ArsB distinct from the previously-characterized DASS family of proteins. The experiments described in the manuscript were carefully carried out, the text was clearly written and the figures well prepared. The work should certainly be seen by the broad readership of Nature Communications.

Here are some minor comments:

1. The authors model that ArsA transfers a substrate to ArsB and helps to increase the transport efficiency is intriguing. Perhaps, the authors would like to include a schematic drawing to illustrate the mechanism?

   The potential interaction between ArsA and ArsB remains an intriguing, yet missing, element of the ATP-coupled metalloid efflux mechanism. In our previous ArsA paper, we attempted to model an ArsA-ArsB complex computationally, but the predictions were not physically plausible. Although limited experimental evidence and reasoning suggest an interaction, we believe that including a schematic could bias assumptions about how these two partners interact. Our AlphaFold3 predictions are referenced in the relevant section of the *Discussion* (line 367). Therefore, we have not added a schematic.

2. The style of the references cited is inconsistent; for some of them the information is incomplete.

   We have inspected each reference to ensure completeness of information and consistent formatting in *Nature* citation style.

3. In Table S1, the model resolution and the B-factor for the model should be included.

   We have added this information to Table S1.
